# Mental health status and the quality of life of infertile women receiving fertility treatment in Bangladesh: A cross-sectional study

**DOI:** 10.1371/journal.pgph.0002680

**Published:** 2023-12-11

**Authors:** A. B. M. Nahid Hasan, Azaz Bin Sharif, Ishrat Jahan, Mosammat Rashida Begum

**Affiliations:** 1 Department of Public Health, North South University, Dhaka, Bangladesh; 2 Infertility Care and Research Centre (ICRC), Dhaka, Bangladesh; Tata Institute of Social Sciences, INDIA

## Abstract

Infertility poses significant physical and psychological challenges for women of reproductive age. In low- and middle-income countries, the prevalence of depression among infertile women is alarmingly high, reaching 44.32%. Additionally, over 50% of infertile women worldwide experience varying degrees of decline in their quality of life. Therefore, this study aimed to assess the effects of infertility on the mental health status and quality of life of infertile women in Bangladesh. Between December 2022 and March 2023, 375 infertile women in Dhaka, Bangladesh were selected using simple random sampling for this cross-sectional study. The participants’ mental health status was assessed using the Depression Anxiety and Stress Scale (DASS-21), while their quality of life was evaluated by the Short Form-12 (SF-12) scale. The prevalence of depression, anxiety, and stress were 59.7%, 55.0%, and 48.7%, respectively. Multiple logistic regression showed that infertile homemakers had 2.98 (95% CI: 1.30 to 6.80) times the odds of depression than government service holders. Aborted infertile women had 1.8 (95% CI: 1.10 to 3.26) times the odds of depression. Infertile women who married between 20 and 24 years old were 49% (95% CI: 0.27 to 0.98) less anxious than those who married earlier. Low-income infertile women (<30,000 BDT) were 2.29 (95% CI: 1.02 to 5.14) times more likely to be stressed than those with higher incomes (>60,000 BDT). Multiple linear regression analysis suggests that education and infertility diagnosis status significantly affect the Mental Component Summary (MCS-12) scores of the Short Form-12 (SF-12). In contrast, age, occupation, and Body Mass Index (BMI) were the significant predictors for the Physical Component Summary (PCS-12). Policymakers may use lessons learned from this study to incorporate appropriate counseling techniques, social awareness campaigns, and media involvement to control the added burden of infertility on women’s psychological health and quality of life.

## Introduction

Infertility is considered a significant public health problem, especially in developing countries where having a child is essential for social, economic, and religious reasons [1]. Infertility is the inability to achieve a clinical pregnancy after 12 months of consistent, unprotected sexual contact [2]. Affecting almost one in five reproductive-age couples, infertility is acknowledged as a social infirmity by WHO, burdening around 48 million couples and 186 million individuals globally [3]. According to the World Fertility Survey (WFS) in South Asian nations, the overall prevalence of infertility is 4%, and for women of reproductive age, it is 15% [4].

Infertility-induced mental health problems are widespread. Previous studies reported that mental health problems among infertile couples are significantly higher than in fertile couples [5, 6]. The negative consequences of infertility on mental health appear in males and females [7], though the significance is more remarkable for females [8]. In low- and middle-income countries, the prevalence of depression among infertile women was 44.32%, while it was 28.03% in high-income nations [9].

Quality of life (QoL) incorporates psychological well-being and physical health status [10]. Infertile women were observed to have a lower quality of life [11]. Infertility and its associated factors negatively impact the quality of life by provoking psychosocial stress, increasing marital conflicts, and decreasing marital satisfaction [12]. More than 50% of infertile women had some degree of impairment in their quality of life [13]. According to B. Rashidi *et al*., educational level and duration of infertility were significant predictors of poorer quality of life of infertile Iranian women [14].

The cultural context of Bangladesh further underscores the significance of the infertility. Infertility from our country’s perspective can be stigmatizing, exacerbating the psychological burden on affected women [15, 16]. The societal pressure to fulfil traditional gender roles as wives and mothers adds an additional layer of complexity, potentially intensifying feelings of inadequacy and despair [17].

In Bangladesh, infertility is still an undervalued problem from both societal and awareness perspectives due to a lack of societal recognition and awareness [18]. The actual state of Infertility in Bangladesh is unknown [18, 19]. Moreover, very little is known about infertile Bangladeshi women’s quality of life and mental well-being. Numerous researches have been conducted around the globe on the mental health and quality of life of infertile women. However, no research emphasizes the extent of the infertility problem in Bangladesh. To the author’s knowledge, prior to this study, there was a dearth of comprehensive research that specifically investigated the mental health and quality of life among infertile women in Bangladesh. While there may have been qualitative studies or limited explorations related to infertility in the country, they did not provide a comprehensive assessment of the mental health and quality of life dimensions in the context of infertility, which was the primary focus of our research.

This research evaluated the mental health status and quality of life of infertile of women receiving infertility treatment. Further, this study intended to assess whether the participants’ background characteristics affected their mental health and quality of life.

## Materials and methods

### Ethical considerations

This research was approved by the Institutional Review Board-IRB of North South University, located in Bashundhara, Dhaka-1229, Bangladesh. A written consent was obtained from all participants. The study’s aims and nature were informed and explained to the respondents before consent was obtained. The Declaration of Helsinki’s principles were followed in this study. The participants’ names and addresses, collected with permission, are stored securely by the authors only, ensuring confidentiality throughout the study. Once the study’s purpose is fulfilled, the data will be securely disposed of to maintain participants’ anonymity and privacy. All data were analyzed anonymously.

### Study population

This cross-sectional study was conducted from December 2022 to March 2023. The inclusion criteria were: (1) Women aged between 18 and 45 years to capture individuals within the reproductive age range. (2) Who has been actively trying to conceive for 12 months or more of regular, unprotected sexual intercourse without achieving a clinical pregnancy [2]. (3) Those participants who were taken treatment from the Infertility Care and Research Center- ICRC. The exclusion criteria were (1) Women who have been infertile for less than three months since diagnosis and disagreed with participating in this study. (2) Women currently undergoing in vitro fertilization (IVF) treatment. (3) Also excluded women who had critical medical illnesses that may independently impact fertility.

### Sample size and sampling

The sample size was calculated using the formula of Cochran’s

$$n=\frac{Z^{2}\;p(1-p)}{e^{2}}.$$

With a 6% of margin of error (e), considering the prevalence of mental distress among infertile Bangladeshi women (p = 62.5%) as reported in a previous study [20], and the standard normal variate of 1.96 (z), the required sample size was 250. We assumed the 10% non-response rate resulting in a sample size of 275. But the study team reached a large sample of 375. A total of 75 participants were excluded from the study as they did not fulfill the interview encompasses both incomplete interviews and instances where participants felt confused or sensitive about certain questions, leading to their non-completion. Finally, 300 study participants who met the inclusion-exclusion criteria completed the interview.

Simple random sampling was applied to select the participants. Patients visiting this hospital had doctor appointments scheduled in advance, and the hospital’s patient care department kept a record of these appointments. We got a serial list of patients from the department and randomly chose five patients each day by their registration id who met our criteria for inclusion and exclusion. Before implementation, the questionnaire was first pre-tested on 5% of the study population.

### Measures

A semi-structured questionnaire was employed to gather the following information:

### Depression anxiety, and stress scale (DASS-21)

The DASS-21 scale is a valid and reliable scale for measuring psychological health. It is a condensed version of the 42-item DASS scale, comprising the depression, anxiety, and stress subscales [21, 22]. There are seven items in each of the three DASS-21 sub-scales. This well-known and widely used DASS-21 scale has been translated and validated in Bengali [23]. On a four-point Likert scale ranging from 0 (never) to 3 (almost always), respondents were questioned about their level of mental distress over the previous four weeks. Individual depression, anxiety, and stress scores were calculated by summing the scores for their respective seven items. The final score for the three dimensions was then multiplied by two to obtain a score between 0 and 42 [21, 24]. Individual scores for these three subscales were then categorized into five severity categories: normal, mild, moderate, severe, and extremely severe [25]. “**Table 1”** describes what score puts an individual into these five categories.

**Table 1:** Recommended cut-off for Depression Anxiety and Stress Scale (DASS-21).

Severity	DASS21-D	DASS21-A	DASS21-S
Normal	0–9	0–7	0–14
Mild	10–13	8–9	15–18
Moderate	14–20	10–14	19–25
Severe	21–27	15–19	26–33
Extremely Severe	28+	20+	34+

A score ranges from 0–42 for each subscale. DASS21-D: Depression sub-scale of The Depression Anxiety and Stress Scales 21items; DASS21-A: Anxiety sub-scale of The Depression Anxiety and Stress Scales 21items; and DASS21-S: Stress sub-scale of The Depression Anxiety and Stress Scales21 items.

### Short form-12 (SF-12) for quality of life

The SF-12 Quality of Life scale is a shorter version of the QoL-36 scale widely used to measure an individual’s quality of life and/or health-related quality of life [26]. It’s a brief, easy-to-use, and robust instrument to measure the quality of life, which was developed by Ware *et al*. in 1995 [27]. Islam *et al*. translated, culturally adapted, and validated the English version of “Short Form SF-12” into Bengali in 2017 [28].

This scale assesses the quality of life in terms of overall health (Item 1), physical function (Items 2 and 3), physical health (Items 4 and 5), physical problems (Items 7 and 6), physical pain (Item 8), social functioning (Item 9), vitality and vital energy (Item 11), and mental health (items 10 and 12). The 12 items are used to derive two summary measures, i.e., physical component summary (PCS) and mental component summary (MCS), each containing six questions [29]. Items are rated on a three to six-point Likert scale; a lower score indicates poor health. Scores of negative items (# 2, 3, 4, 5, 6, 7, 11, and 12) are reversed so that a higher score indicates better health. According to the scoring manual, scores were transformed into the 0 to 100 range. A higher score indicates higher levels of QoL [30, 31]. This study used the mean score for the US population as a cut-off, as no reference score was calculated for the Bangladeshi population. The average PCS-12 and MCS-12 scores for the United States population are 50 (out of 100) [32].

### Additional covariates

Sociodemographic data including age, husbands age, age of marriage, duration of marriage, education, occupation, and Income; Infertility-related questions as: Type of infertility, duration of infertility, infertility diagnosis, treatment duration, abortion history. BMI was calculated by dividing weight in kilograms by the square of height in meters (kg/m2). It was done according to WHO guidelines, defining it as underweight less than 18.5 kg/m2, moderate was 18.5–24.9 kg/m2, overweight was 25.0–29.9 kg/m2, and obese was more than 30 kg/m2. Depression, anxiety, and stress scores were dichotomized for the logistic regression analyses, where individuals belonging to the normal category were recoded as “0”, and the other four (mild, moderate, severe, extremely severe) categories were coded as “1”.

### Statistical analyses

The baseline characteristics of the participants were described with the frequencies and percentages for the categorical variables; the mean and standard deviation for the quantitative variables. The multiple logistic regression model was used to determine the effects of explanatory variables on the individual’s mental health status. There different multiple logistic regression was conducted which is placed in a single Table 4. Candidate explanatory variables for the multiple logistic regression model were determined by assessing their significant bivariate relationship with the outcomes using a chi-square test. The significant explanatory variables included in the multiple logistic regression model were age of marriage, marriage duration, duration of infertility, infertility treatment duration, education, occupation, income, and abortion history.

Multiple linear regression models were used to assess the effect of explanatory variables on the PCS-12 and MCS-12 scores. The Shapiro-Wilk test was used to examine the normality of the PCS-12 and MCS-12 scores. The bivariate relationship between the PCS-12 and MCS-12 scores and the explanatory variables was first explored using the independent sample t-test or One-way Analysis of Variance (ANOVA) techniques depending on the number of categories of the explanatory variables. Only significant explanatory variables were included in the final multiple linear regression model. Analyses were performed using the STATA software (V14 StataCorp LP, TX, USA). The hypothesis tests were two-tailed, with the P value *<* 0.05 considered significant.

## Results

The background characteristics of the respondents are presented in “**Table 2”.** The majority of the participants (42.3%) were aged over 30 years. About 85% of the participants’ husbands were older than 30, with 18.7% above 40 years. Almost one-third of the study subjects were married before 20 years. Three-quarters of the respondents were married for over five years, and almost 60% of the participants have had infertility problems for 4 years or more. Infertility diagnosed for males, females, or both was around 70%, while the rest, 30%, were unknown. Primary infertility accounts for 69.3% of the patients, 73% of the women had no previous history of abortion. More than 50% of the participants had a graduate level of education or higher; however, 63% of the study participants were homemakers. Half of the respondents appeared to be either overweight or obese. Only 17% had an income of more than 60,000 BDT.

**Table 2:** Background characteristics of the infertile patients (n = 300).

Variables	Number (%)
Age^**ǂ**^	
≤25	56 (18.7)
26–30	117 (39.0)
≥31	127 (42.3)
Husbands age^**ǂ**^	
≤30	44 (14.7)
31–35	112 (37.3)
36–40	88 (29.3)
>40	56 (18.7)
Age of marriage ^**ǂ**^	
≤19	98 (32.7)
20–24	97 (32.3)
≥25	105 (35.0)
Duration of marriage^**ǂ**^	
≤4	75 (25.0)
5–8	116 (38.6)
≥9	109 (36.4)
Infertility diagnoses	
Husband	39 (13.0)
Wife	136 (45.3)
Both	33 (11.0)
Unknown	92 (30.7)
Duration of Infertility^**ǂ**^	
≤3	118 (39.3)
4–6	104 (34.7)
≥7	78 (26.0)
Infertility treatment duration ^**ǂ**^
≤3	170 (56.7)
4–6	76 (25.3)
≥7	54 (18.0)
Types of Infertility	
Primary	208 (69.3)
Secondary	92 (30.7)
Education	
HSC and below	121 (40.3)
Graduate	101 (33.7)
Post-Graduate	78 (26.0)
Occupation	
Govt. Job	41 (13.7)
Private Job	57 (19.0)
Homemaker	189 (63.0)
Others	13 (4.3)
Income^b^	
<30000	109 (36.3)
30000–60000	140 (46.7)
>60000	51 (17.0)
Abortion History	
No	219 (73.0)
Yes	81 (27.0)
BMI (kg/m2)	
Underweight	4 (1.3)
Healthy weight	145 (49.4)
Overweight and obese	151 (50.3)

^**ǂ**^In years

^**b**^Bangladeshi Taka-BDT per month, **HSC** = Higher Secondary School certificate (12 Grade), **BMI =** Body Mass Index

**Homemaker** = a wife who manages a household. **other occupations = (**Business, students, freelance jobs).

The Table 3 provides a breakdown of depression, anxiety, and stress levels, revealing a nuanced picture. For depression, while the overall prevalence is 59.7%, the distribution spans from mild (17.7%) to extremely severe (8.7%) depression. Similarly, anxiety’s aggregated prevalence of 55% encompasses levels from mild (7.7%) to extremely severe (15%). Regarding stress, the overall prevalence is 48.7%, encompassing mild (12.3%) to extremely severe (5.0%) cases.

**Table 3:** Mental health status among the infertile women receiving fertility treatment in Bangladesh(n = 300).

	Number (%)
**Depression**	
Normal	121 (40.3)
Mild	53 (17.7)
Moderate	69 (23.0)
Severe	31 (10.3)
Extremely Severe	26 (8.7)
**Anxiety**	
Normal	135 (45.0)
Mild	23 (7.7)
Moderate	73 (24.3)
Severe	24 (8.0)
Extremely Severe	45 (15.0)
**Stress**	
Normal	154 (51.3)
Mild	37 (12.3)
Moderate	54 (18.0)
Severe	40 (13.1)
Extremely Severe	15 (5.0)

Individual scores for three subscales classified into five severity categories: normal, mild, moderate, severe, and extremely severe.

**(“Table 4”)** displays the results of three binary logistic regression analyses where the outcome variables were depression, anxiety, and stress. Homemakers had 2.98 times higher odds of being depressed than the participants who had government jobs as their profession (CI: 1.30 to 6.80). Those with a history of abortion were 1.8 times higher odds of having depression compared to those without an abortion history. Individuals who married at the age of 20–24 were 49% fewer odds of being anxious than those who married at ≤19. Participants who earned less than 30000 BDT per month had 2.29 times higher odds of being stressed than people with more than 60000 BDT per month (CI: 1.02 to 5.14).

**Table 4:** Adjusted effects of explanatory factors on depression, anxiety, and stress.

*(n = 300)*	Depression		Anxiety		Stress	
	OR	95% CI	OR	95% CI	OR	95% CI
Age of marriage ^**ǂ**^						
≤19	.0.73 (Ref)		(Ref)		(Ref)	
20–24	1.18	0.60, 2.31	**0.51**	**0.27, 0.98**	1.15	0.62, 2.16
≥25	1.35	0.62, 2.91	0.51	0.24, 1.08	1.07	0.52, 2.20
Duration of marriage ^**ǂ**^						
≤4	(Ref)		(Ref)		(Ref)	
5–8	0.74	0.34, 1.61	0.54	0.24, 1.17	0.64	0.30, 1.38
≥9	1.16	0.44, 3.04	0.64	0.25, 1.67	0.97	0.38, 2.48
Duration of infertility ^**ǂ**^						
≤3	0.65	0.17, 2.43	0.83	0.23, 2.97	0.78	0.23, 2.67
4–6	0.88	0.28, 2.70	1.38	0.47, 4.06	0.90	0.32, 2.52
≥7	(Ref)		(Ref)		(Ref)	
Infertility treatment						
duration ^**ǂ**^						
≤3	(Ref)		(Ref)		(Ref)	
4–6	0.86	0.39, 1.89	1.68	0.78, 3.58	0.95	0.45, 2.00
≥7	1.22	0.34, 4.33	2.14	0.65, 7.09	0.98	0.31, 3.09
Education						
HSC and below	1.42	0.64, 3.18	0.65	0.29, 1.43	0.96	0.44, 2.07
Graduate	1.25	0.64, 2.47	0.91	0.46, 1.77	0.87	0.45, 1.69
Post-Graduate	(Ref)		(Ref)		(Ref)	
Occupation						
Government Job	(Ref)		(Ref)		(Ref)	
Private Job	1.11	0.46, 2.64	0.94	0.39, 2.25	0.54	0.23, 1.28
Homemaker	**2.98**	**1.30, 6.80**	1.76	0.78, 3.98	0.72	0.32, 1.61
Others	1.58	0.38, 6.45	2.15	0.51, 9.08	1.56	0.37, 6.64
Income^b^						
<30000	0.92	0.40, 2.12	1.34	0.60, 3.02	**2.29**	**1.02, 5.14**
30000–60000	0.84	0.41, 1.73	1.12	0.55, 2.27	1.64	0.81, 3.34
>60000	(Ref)		(Ref)		(Ref)	
Abortion History						
No	(Ref)		(Ref)		(Ref)	
Yes	**1.80**	**1.10, 3.26**	1.40	0.80, 2.46	1.09	0.63, 1.88

Multiple logistic regression model determines explanatory variables’ effects on the individual’s mental health status.

Candidate explanatory variables for the multiple logistic regression model were determined by assessing their significant bivariate relationship with the outcomes using a chi-square test.

**OR =** Odds ratio**, CI =** Confidence interval**, bold** indicates statistical significance (p< 0.05**),**

^**ǂ**^In years

^**b**^Bangladeshi Taka Per Month, **HSC**-Higher Secondary School certificate (12 Grade), **BMI-**Body Mass Index. **Homemaker**- a wife who manages a household, **other occupations (Business, students, freelance jobs), Ref =** Reference group/ category

We conducted a one-way ANOVA analysis to explore differences in SF-12 scores across various background variable categories (“**Table 5”)**. MCS-12 and PCS-12 scores reflect mental and physical health dimensions within the SF-12 scale, contributing to a comprehensive assessment. The overall mean (SD) for Mental Component Summary (MCS-12) and Physical Component Summary (PCS-12) scores were 37.95 (6.93) and 40.7 (6.17), respectively. In relation to MCS-12 scores, statistically significant differences were found across infertility diagnoses (p = 0.017), education (p = 0.001), and occupation (p = 0.001). Similarly, for PCS-12 scores, significant mean differences were observed with respect to occupation (p = 0.005) and BMI categories (p = 0.02)

**Table 5:** Comparison of MCS-12, and PCS-12 scores with the different background variables (*n = 300)*.

				
*PCS-12 (Mean ± SD) 40*.*77 ± 6*.*17*				
*MCS-12(Mean ± SD) 37*.*94 ± 6*.*93*				
	MCS-12	P value	PCS-12	P value
	(Mean ± SD)		(Mean ±SD)	
Age (years)	≤25	38.12 ± 7.83	0.224	42.00 ± 6.67 0.082*
	26–30	38.69 ± 6.93		39.87 ± 5.91
	≥31	37.17 ± 6.47		41.06 ± 6.09
Infertility Diagnoses	Husband	38.33 ± 5.67	**0.017** **	42.27 ± 5.60 0.058*
	Wife	38.78 ± 6.95		40.02 ± 6.04
	Both	34.56 ± 5.87		42.63 ± 6.68
	Unknown	37.75 ± 7.43		40.57 ± 6.24
Education	HSC^**a**^ and below	39.58 ± 6.95	**0.001** **	40.69 ± 5.81 0.952
	Graduate	36.25 ± 6.13		40.72 ± 6.24
	Post Graduate	37.60 ± 7.38		40.96 ± 6.66
Occupation	Government Job	36.00 ± 6.53	**0.001** **	40.05 ± 6.48 0.005**
	Private Job	35.69 ± 7.40		42.49 ± 6.46
	Homemaker	39.16 ± 6.67		40.13 ± 5.89
	Others ǂ	36.30 ± 6.28		44.84 ± 5.44
BMI	Underweight	34.33 ± 10.43	0.067*	46.77 ± 5.04 0.024**
	Healthy Weight	38.79 ± 7.18		40.00 ± 6.78
	Overweight and obese	37.17 ± 6.45		41.35 ± 5.41

**One-way ANOVA** for background variables with more than two categories were conducted**, Independent t-test** for variables with two categories; in this table**, only significant variables are displayed.**

**indicated statistical significance at p< 0.05

*statistical significance at p< 0.10

^**ǂ**^ **Bold** indicates significant at p<. 05.

other occupation = (Business, students, freelance jobs), ^**a**^Higher Secondary School Certificate (12 grade), **Homemaker** = a wife who manages a household, **BMI** = Body Mass Index.

Multiple linear regression analysis were modeled to identify the predictors of the MCS-12 and PCS-12 quality of life scores **(“Table 6”)**. Only the variables significant in the bi-variate relationship were included in the multiple linear regression model. Compared to the education level of high school or lower, the mean MCS-12 scores were significantly lower for those who completed graduation (ß = -2.73; 95% CI: -4.70 to -0.77). The mean MCS-12 scores of participants whose infertility was attributed to both husband and woman problems were, on average, 3.82 points lower than those whose infertility was believed to be due to a husband problem (ß = -3.82; 95% CI: -6.94 to -0.70). Regarding the PCS-12 scores, infertile women aged between 26 and 30 years (ß = -2.38; 95% CI: -4.40 to -0.36) had significantly lower scores than women under 26 years. Mean PCS-12 scores were significantly higher for the infertile women involved in other occupations (Business, students, freelance jobs) than the government job (ß = 4.54; 95% CI: 0.58 to 8.49). Compared to under-weight infertile women, mean PCS-12 scores for healthy-weight infertile women were, on average, 6.36 lower (ß = -6.36, 95% CI: -12.45 to -0.27).

**Table 6:** Factors affecting MCS-12, and PCS-12 scores among the infertile women receiving fertility treatment in Bangladesh.

*(n = 300)*	*MCS-12*		*PCS-12*	
	*β*	*95% CI*	*β*	*95% CI*
Age^a^				
≤25	(ref)		(ref)	
26–30	1.74	[-0.49, 3.97]	**-2.38** **	[-4.40, -0.36]
≥31	0.60	[-1.65, 2.86]	-1.16	[-3.20, 0.88]
Infertility Diagnoses ^a^				
Husband	(ref)		(ref)	
Wife	-0.05	[-2.48, 2.38]	-1.32	[-3.53, 0.87]
Both	**-3.82** **	[-6.94, -0.70]	0.87	[-1.94. 3.69]
Unknown	-0.90	[-3.45, 1.64]	-0.91	[-3.22, 1.39]
Education				
HSC^a^ and below	(ref)		(ref)	
Graduate	**-2.73** **	[-4.70, -0.77]	0.01	[-1.76, 1.78]
Post-Graduate	-1.42	[-3.62, 0.78]	0.77	[-1.22, 2.76]
Occupation				
Government Job	(ref)		(ref)	
Private Job	0.33	[-2.42, 3.08]	2.20*	[-0.28, 4.70]
Homemaker	2.31*	[-0.18, 4.82]	0.23	[-2.02, 2.50]
Others^***ǂ***^	-0.09	[-4.46, 4.27]	**4.54** **	[0.58, 8.49]
BMI				
Underweight	(ref)		(ref)	
Health Weight	5.07	[-1.66, 11.80]	**-6.36** **	[-12.45, -0.27]
Overweight and Obese	3.31	[-3.39, 10.03]	-5.16*	[-11.24, 0.91]

**β =** Standardized (regression) coefficients**, CI =** Confidence interval**. Bold** indicates significant at p <0.0, **ref =** Reference group/ category

Two separate **Multiple linear regression models are** presented in this table, only significant explanatory variables were included in the final multiple linear regression model from the bivariate relationship between the PCS-12, and MCS-12 scores and the explanatory variables (p< 0.10 assumed to pick variable in final model).

**indicated statistical significance at p≤ 0.05

*statistical significance at p< 0.10

^**ǂ**^ other occupation = (Business, students, freelance jobs)

^a^ In years, b Higher Secondary School Certificate (12 grade)

## Discussion

The results of the current study explored the multifaceted mental health and quality of life experiences of infertile Bangladeshi women. With a pioneering spirit, we exposed the high and troubling rates of depression, anxiety, and stress among infertile individuals, highlighting the critical need for specific forms of support and intervention. We find out how important factors like employment status, history of abortion, age of marriage, and socioeconomic background are to the mental health and overall quality of life of infertile women by looking at them in depth. To the best of the authors’ knowledge, this study is the first to explore the effects of infertility on mental health and quality of life among Bangladeshi infertile women.

This research manifests an overall poor psychological status among the respondents regarding depression, anxiety, and stress. Infertility often leads to feelings of inadequacy, isolation, and disrupted self-identity, which, in turn, can trigger a cascade of psychological responses [33]. The profound desire for motherhood, coupled with the societal emphasis on familial roles, can amplify the emotional burden carried by these women [34, 35]. Moreover, the experience of infertility is characterized by a prolonged and often uncertain journey, laden with medical procedures, treatment failures, and dashed hopes. This uncertain trajectory contributes to a heightened state of stress and anxiety, which can lead to an enduring state of heightened stress levels, further exacerbating the psychological strain [36].

According to the current study, abortion history among infertile women has a negative impact on their mental health. The psychological burden carried by women with a history of abortion is exacerbated by the uncertainty surrounding their ability to conceive again. After an abortion, infertile women may give up hope of becoming pregnant again, which can cause stress and guilt on the inside, which is consistent with previous research conducted in Middle Eastern countries [37–39]. The findings of the current research also highlight the importance of marriage age as a key determinant of anxiety levels in women experiencing infertility issues as the late marriage correlates with a significant reduction in anxiety among infertile women and consistently finds a strong inverse relationship with the psychological distress [40–42].

Our findings also revealed that unemployed infertile women who maintain home activities suffer from depression more than those who work in government offices. It is worth noting that government employment opportunities in Bangladesh are widely regarded as being more secured, esteemed, and fulfilling. This perception of job stability and societal regard may potentially play a role in alleviating mental health issues [43]. A study conducted by Fatemeh Ramezanzadeh et al. in Iran found that the educational and employment status of infertile women significantly affected depression, supporting our findings [44].

Our results demonstrated a strong relationship between stress levels and socioeconomic status, particularly for women. Those who earned less than 30,000 Bangladeshi Taka (BDT) were found to be more susceptible to stress than those from the upper-middle and upper classes. These findings aligned with those of Negris et al., who observed a connection between individuals perceiving higher levels of emotional stress and their income [45]. The high cost of infertility treatments, interventions, and medicines is what makes women from lower socioeconomic backgrounds more stressed [46, 47]. Research conducted in Bangladesh by Papreen Nahar highlights how infertility can lead to poverty, particularly among childless families in Bangladesh [19]. It becomes evident that childlessness can result in economic struggles, affecting women’s mobility, men’s income, marriage dynamics, treatment expenses, and access to microcredit [16, 19].

Evidence from the current study reports that an infertile woman leads a relatively low quality of life, and the distressing psychological impact, societal pressure, and potential strain on relationships can collectively contribute to a lower sense of well-being for infertile women. In alignment with prior research findings, the current study underscores the resonance of existing literature [12, 13]. Alarmingly, when infertility was attributed to both husband and wife, had a substantial negative impact on the mental component scores of the quality of life among women in the study sample. This finding is consistent with the trends observed in other study [48], and highlights the pronounced effect of infertility on the mental well-being of women [49]. Infertile women’s educational level and infertility diagnosis status were significantly associated with the mental component score of the quality of life [48, 50].

In the context of the present research, infertile women’s age appeared to have a negative effect on the Physical Component Score (PCS-12). Women in Bangladesh are usually exposed to early marriage, and infertility-related problems may get detected early due to the social pressure of inheriting. An opposite result was observed in Wdowiak *et al*., *who* observed a positive association between age and the overall score on the Fertility QoL, even though they researched in a different socio-economic and cultural context [12]. As indicated by the current study’s results, Dutta et al. reported a notable and positive correlation between the age of women and their perceived quality of life among Indian infertile individuals [51].

The occupation was significant in predicting the physical component scores of quality of life, which was consistent with research conducted in China [52]. Particular occupations might involve physically demanding tasks or require a higher level of physical fitness, which could directly impact an individual’s overall physical well-being [53]. Additionally, increasing body weight was associated with a lower quality of life. The poor health conditions and potential physical limitations of malnourished individuals inhibit their ability to engage in daily activities and thus affect their quality of life [54]. Researchers around the world also compared the quality of life status questionnaires in patients with and without obesity and concluded similar results [26, 55].

Turning our attention to the implications of the result will help us understand the policy implications for improving the mental health and quality of life of infertile women in Bangladesh. Prioritizing mental health services, especially for women who have had past abortions, is crucial. Promoting delayed marriages and providing support during transitional phases can lead to better mental health outcomes. Healthcare providers should routinely assess and address psychological well-being, possibly incorporating mental health screenings into infertility treatment and offering counseling services. Additionally, recognizing the role of employment in reducing depression among infertile women, policymakers should emphasize women’s engagement in work and social activities, facilitating employment opportunities for them to improve their mental health.The following factors strengthen this research: a) interviews were conducted by trained medical students under the supervision of the authors; b) mental health status was assessed along with quality of life; and c) research was carried out at a reputed fertility treatment centre in Bangladesh, where people from every corner of the country come for treatment. A few limitations should be stated when evaluating these results. It is a single-centre interview where samples were taken from only one centre, which may affect the results’ generalizability. Due to the study’s cross-sectional nature, it is impossible to draw a causal inference.

## Conclusion

Infertility negatively affects the mental health status and quality of life of infertile women receiving fertility treatment in Bangladesh. Appropriate counseling techniques, social awareness campaigns, and media involvements may be initiated by the government and/or policymakers to reduce the burden of poor mental health among infertile women, thus ensuring a better quality of life.

## Supporting information

S1 Data (XLS)

S1 Questionnaire (PDF)
