# Correction: Mental health status and the quality of life of infertile women receiving fertility treatment in Bangladesh: A cross-sectional study

**DOI:** 10.1371/journal.pgph.0004271

**Published:** 2025-01-30

**Authors:** The PLOS GLOBAL PUBLIC HEALTH Staff

In Table 4, there is an error in the odds ratio (OR) column under depression in the first category of “age of marriage” variable. The correct entry should be “Ref” instead of “.0.73 Ref.”.

Consequently, the Table 5 is formatted incorrectly. The mean (sd) and p values for both pcs-12 and mcs-12 are merged into a single column.

Moreover, in Table 6, some cells are inadvertently left-aligned. Please see the correct Tables 4, 5 and 6 here.

**Table 4:** Adjusted effects of explanatory factors on Depression, Anxiety, and Stress.

*(n = 300)*	Depression		Anxiety		Stress	
	OR	95% CI	OR	95% CI	OR	95% CI
Age of marriage ^**ǂ**^						
≤19	(Ref)		(Ref)		(Ref)	
20–24	1.18	0.60, 2.31	**0.51**	**0.27, 0.98**	1.15	0.62, 2.16
≥25	1.35	0.62, 2.91	0.51	0.24, 1.08	1.07	0.52, 2.20
Duration of marriage ^**ǂ**^						
≤4	(Ref)		(Ref)		(Ref)	
5–8	0.74	0.34, 1.61	0.54	0.24, 1.17	0.64	0.30, 1.38
≥9	1.16	0.44, 3.04	0.64	0.25, 1.67	0.97	0.38, 2.48
Duration of infertility ^**ǂ**^						
≤3	0.65	0.17, 2.43	0.83	0.23, 2.97	0.78	0.23, 2.67
4–6	0.88	0.28, 2.70	1.38	0.47, 4.06	0.90	0.32, 2.52
≥7	(Ref)		(Ref)		(Ref)	
Infertility treatment duration ǂ						
≤3	(Ref)		(Ref)		(Ref)	
4–6	0.86	0.39, 1.89	1.68	0.78, 3.58	0.95	0.45, 2.00
≥7	1.22	0.34, 4.33	2.14	0.65, 7.09	0.98	0.31, 3.09
Education						
HSC and below	1.42	0.64, 3.18	0.65	0.29, 1.43	0.96	0.44, 2.07
Graduate	1.25	0.64, 2.47	0.91	0.46, 1.77	0.87	0.45, 1.69
Post-Graduate	(Ref)		(Ref)		(Ref)	
Occupation						
Government Job	(Ref)		(Ref)		(Ref)	
Private Job	1.11	0.46, 2.64	0.94	0.39, 2.25	0.54	0.23, 1.28
Homemaker	**2.98**	**1.30, 6.80**	1.76	0.78, 3.98	0.72	0.32, 1.61
Others	1.58	0.38, 6.45	2.15	0.51, 9.08	1.56	0.37, 6.64
Income^b^						
<30000	0.92	0.40, 2.12	1.34	0.60, 3.02	**2.29**	**1.02, 5.14**
30000–60000	0.84	0.41, 1.73	1.12	0.55, 2.27	1.64	0.81, 3.34
>60000	(Ref)		(Ref)		(Ref)	
Abortion History						
No	(Ref)		(Ref)		(Ref)	
Yes	**1.80**	**1.10, 3.26**	1.40	0.80, 2.46	1.09	0.63, 1.88

Multiple logistic regression model determines explanatory variables’ effects on the individual’s mental health status.

Candidate explanatory variables for the multiple logistic regression model were determined by assessing their significant bivariate relationship with the outcomes using a chi-square test.

**OR =** Odds ratio**, CI =** Confidence interval**, bold** indicates statistical significance (p< 0.05**),** ^**ǂ**^In years, ^**b**^Bangladeshi Taka Per Month, **HSC**-Higher Secondary School certificate (12 Grade), **BMI-**Body Mass Index. **Homemaker**- a wife who manages a household, **other occupations (Business, students, freelance jobs), Ref =** Reference group/ category

**Table 5:** Comparison of MCS-12, and PCS-12 scores with the different background variables (*n = 300)*.

*PCS-12 (Mean ± SD) = 40*.*77 ± 6*.*17*					
*MCS-12(Mean ± SD) = 37*.*94 ± 6*.*93*					
		MCS-12 (Mean ± SD)	P value	PCS-12 (Mean ± SD)	P value
	≤25	38.12 ± 7.83		42.00 ± 6.67	
Age (years)	26–30	38.69 ± 6.93	0.224	39.87 ± 5.91	0.082*
	≥31	37.17 ± 6.47		41.06 ± 6.09	
	Husband	38.33 ± 5.67		42.27 ± 5.60	
	Wife	38.78 ± 6.95		40.02 ± 6.04	
Infertility Diagnoses	Both	34.56 ± 5.87	**0.017** **	42.63 ± 6.68	0.058*
	Unknown	37.75 ± 7.43		40.57 ± 6.24	
	HSC^**a**^ and below	39.58 ± 6.95		40.69 ± 5.81	
Education	Graduate	36.25 ± 6.13	**0.001** **	40.72 ± 6.24	0.952
	Post Graduate	37.60 ± 7.38		40.96 ± 6.66	
	Government Job	36.00 ± 6.53		40.05 ± 6.48	
	Private Job	35.69 ± 7.40		42.49 ± 6.46	
Occupation	Homemaker	39.16 ± 6.67	**0.001** **	40.13 ± 5.89	**0.005** **
	Others **ǂ**	36.30 ± 6.28		44.84 ± 5.44	
	Underweight	34.33 ± 10.43		46.77 ± 5.04	
BMI	Healthy Weight	38.79 ± 7.18	0.067*	40.00 ± 6.78	**0.024** **
	Overweight and obese	37.17 ± 6.45		41.35 ± 5.41	

**One-way ANOVA** for background variables with more than two categories were conducted**, Independent t-test** for variables with two categories; in this table**, only significant variables are displayed.**

**indicated statistical significance at p< 0.05**,**

*statistical significance at p< 0.10**,**

^**ǂ**^ **Bold** indicates significant at p<. 05. other occupation = (Business, students, freelance jobs),

^**a**^Higher Secondary School Certificate (12 grade), **Homemaker** = a wife who manages a household, **BMI** = Body Mass Index.

**Table 6:** Factors affecting MCS-12, and PCS-12 scores among the infertile women receiving fertility treatment in Bangladesh.

*(n = 300)*	*MCS-12*		*PCS-12*	
	*β*	*95% CI*	*β*	*95% CI*
Age^a^				
≤25	(ref)		(ref)	
26–30	1.74	[-0.49, 3.97]	**-2.38** **	[-4.40, -0.36]
≥31	0.60	[-1.65, 2.86]	-1.16	[-3.20, 0.88]
Infertility Diagnoses ^a^				
Husband	(ref)		(ref)	
Wife	-0.05	[-2.48, 2.38]	-1.32	[-3.53, 0.87]
Both	**-3.82** **	[-6.94, -0.70]	0.87	[-1.94. 3.69]
Unknown	-0.90	[-3.45, 1.64]	-0.91	[-3.22, 1.39]
Education				
HSC^a^ and below	(ref)		(ref)	
Graduate	**-2.73** **	[-4.70, -0.77]	0.01	[-1.76, 1.78]
Post-Graduate	-1.42	[-3.62, 0.78]	0.77	[-1.22, 2.76]
Occupation				
Government Job	(ref)		(ref)	
Private Job	0.33	[-2.42, 3.08]	2.20*	[-0.28, 4.70]
Homemaker	2.31*	[-0.18, 4.82]	0.23	[-2.02, 2.50]
Others^***ǂ***^	-0.09	[-4.46, 4.27]	**4.54** **	[0.58, 8.49]
BMI				
Underweight	(ref)		(ref)	
Health Weight	5.07	[-1.66, 11.80]	**-6.36** **	[-12.45, -0.27]
Overweight and Obese	3.31	[-3.39, 10.03]	-5.16*	[-11.24, 0.91]

***β =*** *Standardized (regression) coefficients***, *CI =*** *Confidence interval***. *Bold*** *indicates significant at p <0*.*0*, ***ref =*** *Reference group/ category*

Two separate **Multiple linear regression models are** presented in this table, only significant explanatory variables were included in the final multiple linear regression model from the bivariate relationship between the PCS-12, and MCS-12 scores and the explanatory variables (p< 0.10 assumed to pick variable in final model).

**indicated statistical significance at p≤ 0.05

*statistical significance at p< 0.10

^**ǂ**^ other occupation = (Business, students, freelance jobs)

^a^ In years, b Higher Secondary School Certificate (12 grade)

The publisher apologizes for the errors.
