# Supplementary material for: Mental health status and the quality of life of infertile women receiving fertility treatment in Bangladesh: A cross-sectional study
Source: PLOS Glob Public Health. 2023 Dec 11;3(12):e0002680. doi: 10.1371/journal.pgph.0002680 (PMC10712886; doi:10.1371/journal.pgph.0002680)
Supplement: S1 Questionnaire — (PDF) [file pgph.0002680.s002.pdf]

Identifying Information:

Patient Name: ....

Husband's Name………………………………………………………………

Present Address……………………………………………………………..

Patient's Phone/ Mobile No: ....

Name of the fertility center/ clinic/ hospital……………………………………

SECTION 01_A: BACKGROUND CHARECTERISTICS

Sl No	Entitle	
Q 01	**Age**	…………Years
Q 02	**Husbands' Age**	…………Years
Q 03	**Duration of Marriage**	-----------Years/ --------Months
Q 04	**Length of time infertile**	------------Years/-------Months
Q 05	**Infertility problem in**	1. Husband<br>2. Wife<br>3. Both<br>4. Unknown
Q 06	**Do you have any children? (To identify the secondary infertility)**	1. No<br>2. Yes
Q 07	**Education level**	1. HSC and below<br>2. Graduate<br>3. Post Graduations
Q 08	**Occupation**	1. Govt. Job<br>2. Private Job<br>3. Homemaker<br>4. Others
Q 09	**Household Monthly Income (BDT/M)**	1. Below 30,000<br>2. 30,000-60,000<br>3. More 60,000
Q 10	**Height**	In Centimeters/Inches(cm/inch) …………….
Q 11	**Weight**	In Pounds/Kilograms (lb./kg): ....
Q 12	**Do you have any abortion history? (Spnt)**	1. No<br>2. Yes

SECTION 02: DEPRESSION, ANXIETY AND STRESS SCALE - 21 ITEMS (DASS-21) _Q. 13-33

Please read each statement and circle a number 0, 1, 2, or 3 which indicates how much the statement applied to you ***over the pastweek***. There are no right or wrong answers. Do not spend too much time on any statement. *The rating scale is as follows:*

0 Did not apply to me at all
1 Applied to me to some degree, or some of the time
2 Applied to me to a considerable degree, or a good part of the time
3 Applied to me very much, or most of the time

1	I found it hard to wind down	0	1	2	3
2	I was aware of dryness of my mouth	0	1	2	3
3	I couldn’t seem to experience any positive feeling at all	0	1	2	3
4	I experienced breathing difficulty (ex. excessively rapid breathing, breathlessness in the absence of physical exertion)	0	1	2	3
5	I found it difficult to work up the initiative to do things	0	1	2	3
6	I tended to over-react to situations	0	1	2	3
7	I experienced trembling (ex. in the hands)	0	1	2	3
8	I felt that I was using a lot of nervous energy	0	1	2	3
9	I was worried about situations in which I might panic and make a fool ofmyself	0	1	2	3
10	I felt that I had nothing to look forward to	0	1	2	3
11	I found myself getting agitated	0	1	2	3
12	I found it difficult to relax	0	1	2	3
13	I felt down-hearted and blue	0	1	2	3
14	I was intolerant of anything that kept me from getting on with what I was doing	0	1	2	3
15	I felt I was close to panic	0	1	2	3
16	I was unable to become enthusiastic about anything	0	1	2	3
17	I felt I wasn’t worth much as a person	0	1	2	3
18	I felt that I was rather touchy	0	1	2	3
19	I was aware of the action of my heart in the absence of physical exertion (ex:sense of heart rate increase, heart missing a beat)	0	1	2	3
20	I felt scared without any good reason	0	1	2	3
21	I felt that life was meaningless	0	1	2	3

SECTION_3: SF-12 FOR QUALITY OF LIFE MEASUREMENT

This survey asks for your views about your health. This information will help keep track of how you feel and how well you can do your usual activities. **Answer each question by choosing just one answer**. If you are unsure how to answer a question, please give the best answer you can.

**01**	In general, would you say your health is:	□ 1 Excellent<br>□ 2 Very good<br>□ 3 Good<br>□ 4 Fair<br>□ 5 Poor

The following two questions are about activities you might do during a typical day. Does YOUR HEALTH NOWLIMIT YOU in these activities? If so, how much?

		**Yes, Limited a lot**	**Yes, limited a little**	**No, not limsited at all**
**02**	Moderate activities such as moving a table, pushinga vacuum cleaner, bowling, or playing golf.	□ 1	□ 2	□ 3
**03**	Climbing several flights of stairs.	□ 1	□ 2	□ 3

During the PAST 4 WEEKS have you had any of the following problems with your work or other regular activities AS A RESULT OF YOUR PHYSICAL HEALTH?

**04**	Accomplished less than you would like.	□ 1 YES	□ 2 NO
**05**	Were limited in the kind of work or other activities.	□ 1 YES	□ 2 NO

During the past 4 weeks, have you had any of the following problems with your work or other regular dailyactivities as a result of any emotional problems (such as feeling depressed or anxious)?

**06**	Accomplished less than you would like.	□ 1 YES	□ 2 NO
**07**	7. Did work or activities less carefully than usual.	□ 1 YES	□ 2 NO
**08**	During the past 4 weeks, how much did pain interfere withyour normal work (including work outside the home and housework)?	□ 1 Not at all<br>□ 2 A little bit<br>□ 3 Moderately<br>□ 4 Quite a bit<br>□ 5 Extremely	

These questions are about how you have been feeling during the past 4 weeks. For each question, please give the one answer that comes closest to the way you have been feeling.
How much of the time during the past 4 weeks?

09 Have you felt calm & peaceful?

**All ofthe time**	**Most ofthe time**	**A good bit of the time**	**Some ofthe time**	**A little of the time**	**None of thetime**
□ 1	□ 2	□ 3	□ 4	□ 5	□ 6

10 Did you have a lot of energy?

**All of the time**	**Most of the time**	**A good bit of the time**	**Some of the time**	**A little of the time**	**None of thetime**
□ 1	□ 2	□ 3	□ 4	□ 5	□ 6

11 Have you felt down-hearted and blue?

**All of thetime**	**Most of thetime**	**A good bit ofthe time**	**Some of thetime**	**A little of the time**	**None of thetime**

□ 1	□ 2	□ 3	□ 4	□ 5	□ 6

12 **During the past 4 weeks, how much of the time has your physical health or emotional problemsinterfered with your social activities (like visiting friends, relatives, etc.)?**

All of the time	Most of the time	Some of the time	A little of the time	None of the time
□ 1	□ 2	□ 3	□ 4	□ 5
